# Overexpression of the rice BAHD acyltransferase AT10 increases xylan-bound *p*-coumarate and reduces lignin in *Sorghum bicolor*

**DOI:** 10.1186/s13068-021-02068-9

**Published:** 2021-11-20

**Authors:** Yang Tian, Chien-Yuan Lin, Joon-Hyun Park, Chuan-Yin Wu, Ramu Kakumanu, Venkataramana R. Pidatala, Khanh M. Vuu, Alberto Rodriguez, Patrick M. Shih, Edward E. K. Baidoo, Stephen Temple, Blake A. Simmons, John M. Gladden, Henrik V. Scheller, Aymerick Eudes

**Affiliations:** 1 Joint BioEnergy Institute, Emeryville, CA 94608 USA; 2 Environmental Genomics and Systems Biology Division, Lawrence Berkeley National Laboratory, Berkeley, CA 94720 USA; 3 Forage Genetics International, West Salem, WI 54669 USA; 4 Biological Systems and Engineering Division, Lawrence Berkeley National Laboratory, Berkeley, CA 94720 USA; 5 Department of Biomaterials and Biomanufacturing, Sandia National Laboratories, Livermore, CA 94551 USA; 6 Department of Plant and Microbial Biology, University of California-Berkeley, Berkeley, CA 94720 USA

**Keywords:** Sorghum, Bioenergy, Lignin, Xylan, Ferulate, *p*-Coumarate, Diferulates, Saccharification

## Abstract

**Background:**

The development of bioenergy crops with reduced recalcitrance to enzymatic degradation represents an important challenge to enable the sustainable production of advanced biofuels and bioproducts. Biomass recalcitrance is partly attributed to the complex structure of plant cell walls inside which cellulose microfibrils are protected by a network of hemicellulosic xylan chains that crosslink with each other or with lignin via ferulate (FA) bridges. Overexpression of the rice acyltransferase OsAT10 is an effective bioengineering strategy to lower the amount of FA involved in the formation of cell wall crosslinks and thereby reduce cell wall recalcitrance. The annual crop sorghum represents an attractive feedstock for bioenergy purposes considering its high biomass yields and low input requirements. Although we previously validated the OsAT10 engineering approach in the perennial bioenergy crop switchgrass, the effect of *OsAT10* expression on biomass composition and digestibility in sorghum remains to be explored.

**Results:**

We obtained eight independent sorghum (*Sorghum bicolor* (L.) Moench) transgenic lines with a single copy of a construct designed for *OsAT10* expression. Consistent with the proposed role of OsAT10 in acylating arabinosyl residues on xylan with *p*-coumarate (*p*CA), a higher amount of *p*-coumaroyl-arabinose was released from the cell walls of these lines upon hydrolysis with trifluoroacetic acid. However, no major changes were observed regarding the total amount of *p*CA or FA esters released from cell walls upon mild alkaline hydrolysis. Certain diferulate (diFA) isomers identified in alkaline hydrolysates were increased in some transgenic lines. The amount of the main cell wall monosaccharides glucose, xylose, and arabinose was unaffected. The transgenic lines showed reduced lignin content and their biomass released higher yields of sugars after ionic liquid pretreatment followed by enzymatic saccharification.

**Conclusions:**

Expression of *OsAT10* in sorghum leads to an increase of xylan-bound *p*CA without reducing the overall content of cell wall FA esters. Nevertheless, the amount of total cell wall *p*CA remains unchanged indicating that most *p*CA is ester-linked to lignin. Unlike other engineered plants overexpressing *OsAT10* or a phylogenetically related acyltransferase with similar putative function, the improvements of biomass saccharification efficiency in sorghum *OsAT10* lines are likely the result of lignin reductions rather than reductions of cell wall-bound FA. These results also suggest a relationship between xylan-bound *p*CA and lignification in cell walls.

**Supplementary Information:**

The online version contains supplementary material available at 10.1186/s13068-021-02068-9.

## Background

Lignocellulosic biomass is a potential significant carbon–neutral source of renewable sugars and aromatics for conversion into advanced biofuels and other bioproducts using engineered microorganisms [1]. Among other crop candidates, sorghum is an annual C4 grass that represents an ideal bioenergy feedstock due to its low input requirements, high water use efficiency, efficient nitrogen recycling, and high biomass yields [2, 3]. Optimizing sorghum biomass composition is desired for bioenergy applications since releasing sugars and aromatics at low cost from lignocellulose appears detrimental for developing economically sustainable biorefineries [4]. However, the inherent structure of lignocellulose confers recalcitrance to degradation due to the rigid and compact structure of plant cell walls, which consist of cellulose microfibrils surrounded by a network of interconnected hemicellulose and lignin polymers [5].

**Fig. 1:**
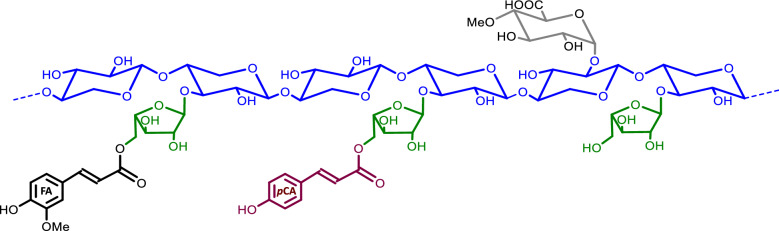
Schematic representation of a glucuronoarabinoxylan (GAX) chain. The xylan backbone is shown in blue. Arabinosyl units (green) can be substituted with ferulate (FA) or *p*-coumarate (*p*CA). A methylated glucuronic acid unit is shown (grey). The rice OsAT10 transferase used in the work is involved in the attachment of *p*CA on GAX

The dominant hemicellulose in grasses is termed glucuronoarabinoxylan (GAX) and consists of a xylan backbone substituted with glucuronic acid and arabinose residues [6]. In addition, the aromatics ferulate (FA) and *p*-coumarate (*p*-CA) are occasionally linked to arabinosyl units (Fig. 1). Feruloyl-arabinose (FA-Ara) residues are typically more frequent than coumaroyl-arabinose (*p*CA-Ara) residues, and substitution patterns also vary depending on grass species and plant organs [7, 8]. These grass-specific FA and *p*CA esters have been implicated in GAX crosslinks that reinforce cell walls [9]. In particular, GAX chains can be cross-linked by FA dimers formed via radical coupling [10, 11], and FA esters also provide nucleation sites for lignification and the cross-linking of GAX to lignins [12, 13]. *p*CA does not undergo oxidative coupling but its involvement in GAX crosslinking via photochemical dimerization has been suggested [14]. In fact, *p*CA is predominantly found ester-linked to lignin in grass cell walls, and it may assist lignin formation by providing a transfer mechanism to optimize the radical coupling of monolignols [15, 16].

Several BAHD acyltransferases that presumably use *p*-coumaroyl-CoA and feruloyl-CoA as donor substrates have been implicated in GAX *p*-coumaroylation and feruloylation, respectively. For example, silencing of *BdAT1* and *SvBAHD01* results in reductions of FA esters on xylan in *Brachypodium distachyon* and *Setaria viridis* [17, 18]. As for *p*CA transferases, overexpression of *OsAT10* in rice results in higher amount of *p*CA-Ara in young leaves, whereas grain cell walls from barley genotypes mutated in *HvAT10* show approximately one-third less *p*CA than wild-type genotypes [19, 20]. Similarly, silencing of *SvBAHD05* in *S. viridis* reduces *p*CA-Ara content, while overexpression of sugarcane *ScAT10* increases *p*CA-Ara in maize [21, 22]. Interestingly, overexpression of these putative *p*-coumaroyl-CoA GAX transferase AT10 genes is often accompanied with enhancements of cell wall saccharification efficiency, which has promoted their use to improve biomass quality in bioenergy crops [22, 23]. Such reductions of cell wall recalcitrance have been tentatively linked to a decrease of cell wall-bound FA measured in plants overexpressing AT10 transferases [19, 22, 23]. As previously hypothesized, this effect on FA esters might occur if *p*-coumaroyl-CoA and feruloyl-CoA compete for transfer onto a shared acceptor (e.g., UDP-arabinose) prior to incorporation into xylan chains such that increase of *p*CA transfer leaves less free acceptor available for FA transfer activity. Alternatively, since *p*-coumaroyl-CoA is a metabolic precursor to feruloyl-CoA, a higher amount of *p*-coumaroyl-CoA recruited for xylan acylation may indirectly affect the pool of feruloyl-CoA available for such activity [20]. No effects on lignin monomeric composition and/or lignin content have been observed in rice, switchgrass, and maize lines expressing *AT10* transferases [19, 22, 23].

In this work, we report on the expression of rice AT10 transferase in sorghum. Engineered sorghum lines show large increases of *p*CA-Ara residues on GAX but no change in total amount of cell wall-bound *p*CA and FA, albeit certain diFAs are increased in some lines. Modified lines display reductions of lignin content, which leads to reduced cell wall recalcitrance and improvement of enzymatic saccharification efficiencies after biomass pretreatment.

## Results

### Generation of sorghum *pSbUbi:AT10* lines expressing *OsAT10*

**Fig. 2:**
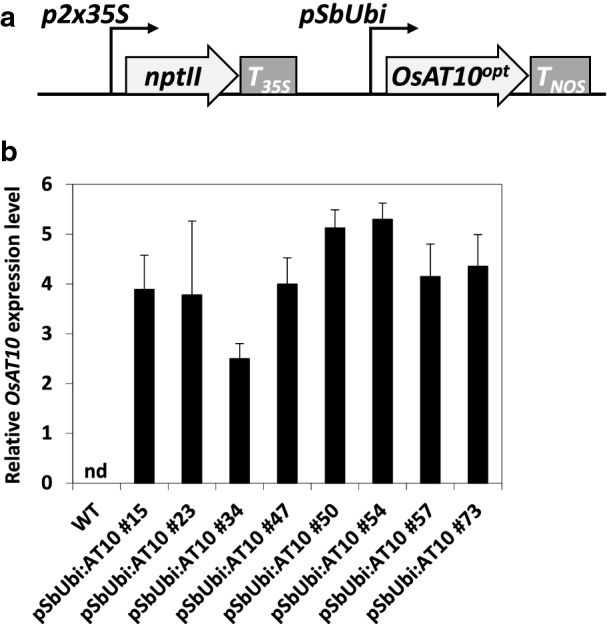
Expression of *OsAT10* in sorghum. Schematic diagram of the construct used for sorghum transformation (**a**) and relative *OsAT10* expression levels among eight independent transgenic lines (**b**). The T-DNA contains a selection marker for kanamycin resistance (*nptII*) under the control of a duplicated 35S promoter (*p2* × *35S*) from cauliflower mosaic virus (CaMV) and harbors the *OsAT10*^*opt*^ gene sequence under the control of the promoter of a sorghum polyubiquitin gene (*pSbUbi*). *T*_*35S*_ and *T*_*NOS*_ denote the terminators from the CaMV 35S and *Agrobacterium tumefaciens* nopaline synthase genes, respectively. *OsAT10* transcripts were detected using RT-qPCR. *OsAT10* expression levels relative to that of the *PP2A* gene are shown. cDNA obtained from wild-type segregants were used as negative control. Values are means ± SD of four biological replicates (*n* = 4). nd, not detected

A construct consisting of an *OsAT10* open reading frame codon-optimized for expression in sorghum and placed downstream of the promoter of a sorghum polyubiquitin gene was built for *Agrobacterium*-mediated sorghum transformation (Fig. 2a). Eighty primary T0 transformants regenerated from calli were analyzed by real-time PCR using extracted gDNA and primers specific to *OsAT10* and to the *nptII* selection marker to identify lines containing a single copy of the transgene. Eight of these single-copy events were grown in the T1 generation and screened by real-time PCR to identify wild-type segregants and homozygous plants (data not shown). T2 wild-type segregant seeds from each line were pooled and used as controls for further experiments. *OsAT10* expression in each transgenic line was validated by qPCR performed on cDNA synthesized from total RNAs obtained from the stems of 3-week-old plants in the T2 generation (Fig. 2b). Measurements of growth parameters including the number of days to panicle emergence, number of flowering tillers, height of the main tiller, stover dry weight, and estimated seeds dry weight did not reveal any consistent differences between wild type and the transgenic lines, showing that *OsAT10* expression has little impact on development in plants grown under controlled conditions (Additional file 1: Table S1).

### *OsAT10* sorghum lines show increased xylan-bound *p*-coumarate

**Fig. 3:**
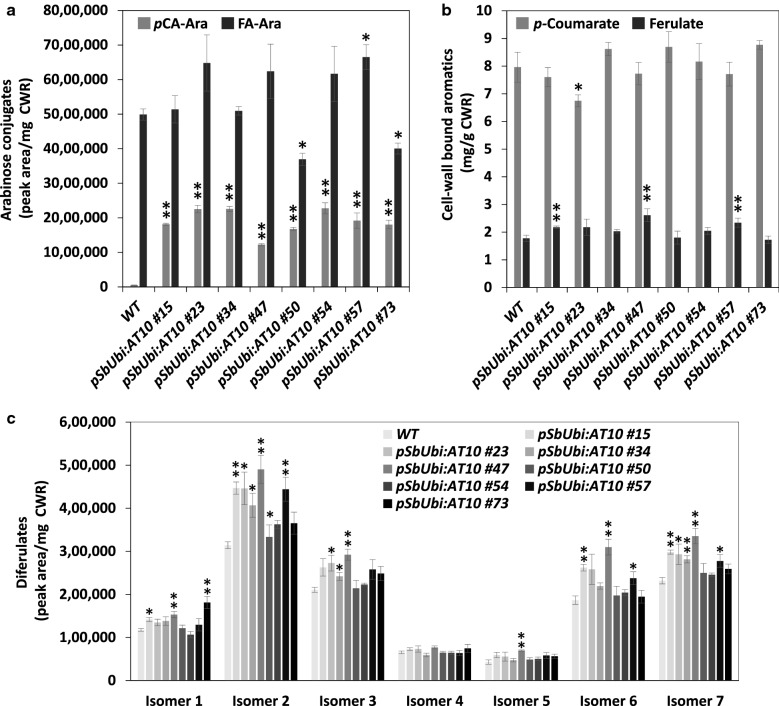
Cell wall-bound aromatics in *pSbUbi:AT10* lines and wild-type segregants (WT). *p*CA-Ara and FA-Ara were released by TFA hydrolysis (**a**). Total *p*-coumarate and ferulate (**b**) and diferulates (**c**) were released by mild alkaline hydrolysis. Values are means ± SE of four biological replicates (*n* = 4). Asterisks indicate significant differences from the WT using the unpaired Student’s *t* test (*P < 0.05, **P < 0.01)

OsAT10 is a putative *p*CA transferase that acylates arabinosyl sidechains on GAX. We used trifluoroacetic acid (TFA) hydrolysis to release *p*-coumaroylated (*p*CA-Ara) and feruloylated (FA-Ara) arabinose units from cell wall residues (CWR) obtained from wild type and *pSbUbi:AT10* transgenic lines. HPLC–ESI–TOF–MS analysis of hydrolysates identified two peaks with accurate mass measurements and fragmentation patterns corresponding to *p*CA-Ara (*m*/*z* = 295) and FA-Ara (*m*/*z* = 325), respectively (Additional file 1: Fig. S1). Integration of peak areas showed a 23–42-fold increase of *p*CA-Ara in *pSbUbi:AT10* lines compared to wild type, whereas FA-Ara was modestly increased by 25% in one line and reduced by 20% and 26% in two other lines (Fig. 3a). Measurements of total cell wall-bound *p*CA and FA released upon mild alkaline hydrolysis of CWR showed reduction of *p*CA (− 15%) in only one line (*pSbUbi:AT10 #23*) and significant increases of FA (+ 18–32%) in three lines compared to control (Fig. 3b). HPLC–ESI–TOF–MS analysis of the alkaline hydrolysates enabled detection of seven peaks with ions at *m*/*z* 295 matching those of FA dimers (Additional file 1: Fig. S2). Compared to wild type, none of the transgenic lines showed reduction of these diFA isomers. Instead, some lines showed increases in isomer 1 (+ 17–35%), isomer 2 (+ 13–35%), isomer 3 (+ 13–28%), isomer 5 (48%), isomer 6 (+ 22–40%), and isomer 7 (+ 18–31%) (Fig. 3c).

### *OsAT10* sorghum lines exhibit reduced lignin

A two-step hydrolysis of cell wall residues (i.e., Klason method) was used to quantify lignin and major cell wall monosaccharides in the *pSbUbi:AT10* lines. Compared to wild-type control, no changes in glucose, xylose, and arabinose could be observed in cell walls from engineered lines, with the exception of one line that had 9% more xylose and another one that showed 18% more arabinose. These results demonstrate that *OsAT10* expression does not result in major changes in cellulose and xylan content (Table 1). Conversely, all transgenic lines showed small reductions of lignin content that ranged between 6 and 16% and were significant (*P* < 0.05) for six out of eight lines compared to wild type (Table 1). Analysis of lignin monomeric composition using pyrolysis coupled to gas chromatography mass spectrometry revealed reductions in the ratio of syringyl (S) to guaiacyl (G) units (S/G) for six of the transgenic lines compared to control (Tables 1 and Additional file 1: Table S2).

**Table 1:** Chemical composition and lignin S/G of cell wall residues (CWR) obtained from stem biomass of wildtype and *pSbUbi:AT10* lines

	Average (mg/g CWR)				Lignin S/G
	Glucose	Xylose	Arabinose	Lignin	
Wildtype	363.1 (10.5)	245.2 (5.5)	27.3 (1.4)	162.0 (3.8)	0.74 (0.03)
*pSbUbi:AT10 #15*	365.3 (5.7)	257.8 (3.1)	28.1 (0.5)	152.5 (8.9)	0.66 (0.03)*
*pSbUbi:AT10 #23*	365.2 (2.6)	268.6 (7.0)*	31.8 (1.7)	134.8 (5.7)*	0.54 (0.04)*
*pSbUbi:AT10 #34*	357.6 (5.1)	255.7 (5.9)	28.1 (0.3)	150.2 (3.6)*	0.45 (0.01)*
*pSbUbi:AT10 #47*	356.9 (12.1)	252.3 (5.8)	33.1 (0.7)*	135.6 (6.2)*	0.64 (0.03)*
*pSbUbi:AT10 #50*	361.1 (5.6)	242.6 (4.6)	27.0 (2.3)	149.6 (7.1)	0.62 (0.01)*
*pSbUbi:AT10 #54*	357.0 (11.9)	258.4 (6.6)	28.1 (0.5)	144.6 (4.9)*	0.62 (0.04)*
*pSbUbi:AT10 #57*	367.0 (6.0)	263.9 (7.6)	28.8 (1.6)	140.2 (6.0)*	0.77 (0.02)
*pSbUbi:AT10 #73*	357.1 (11.7)	256.0 (7.4)	33.0 (2.0)	152.6 (3.2)*	0.77 (0.04)

Values in brackets are the SE from four biological replicates (*n* = 4)

Asterisks indicate a significant difference from the wildtype using the unpaired Student’s *t* test (*P < 0.05)

### *OsAT10* sorghum biomass shows improved saccharification

**Fig. 4:**
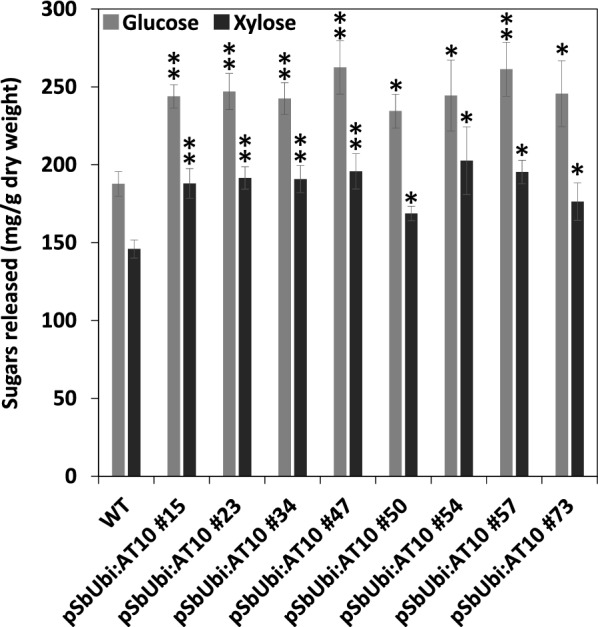
Biomass saccharification of sorghum lines containing the *pSbUbi:AT10* construct. Amounts of glucose and xylose released after ionic liquid pretreatment followed by 72 h of enzymatic digestion with a cellulase and hemicellulose cocktail are shown. Values are means ± SE of four biological replicates (*n* = 4). Asterisks indicate significant differences from wild-type controls (WT) using the unpaired Student’s *t* test (*P < 0.05, **P < 0.01)

The recalcitrance of *OsAT10* engineered sorghum towards enzymatic degradation was evaluated by measuring the amount of sugars released from the biomass after pretreatment with the ionic liquid cholinium phosphate, followed by a 72-h enzymatic hydrolysis using a commercial glycoside hydrolase enzyme mixture (Novozymes Cellic® CTec3 and HTec3). As shown in Fig. 4, a higher sugar titer was obtained for all *pSbUbi:AT10* lines compared to the wild-type control, with significant increases ranging between 25–40% for glucose and 17–39% for xylose. Considering the similar content of the total monosaccharides present in the cell walls of the transgenics and controls (Table 1), we conclude that the modified cell walls of the engineered lines enable more efficient biomass pretreatment and saccharification.

## Discussion

In this work, we report on the successful translation of the *OsAT10* genetic engineering approach to sorghum; concomitant increase of xylan acylation with *p*CA and decrease of lignin content in engineered plants was accompanied with up to 28% increases in the yield of monosaccharides released upon biomass pretreatment and enzymatic saccharification. In stems from wild-type sorghum, little amount of *p*CA-Ara was released in comparison to the amount of FA-Ara after mild acid hydrolysis of CWR. Assuming comparable response factors for *p*CA-Ara and FA-Ara measured under our LC–MS conditions, we can estimate that *p*-coumaroylation of arabinosyl residues on GAX represent ~ 1% of total arabinose acylation (i.e., feruloylation being more frequent). This is similar to previous values obtained with maize stems showing ratios of acylation of arabinose units by *p*CA in the range of 2.5% or less [7, 22]. In sorghum, acyltransferases responsible for xylan *p*-coumaroylation and feruloylation are still unknown, but several gene candidates have been proposed based on their higher expression levels in elongating internodes [24]. In sugarcane, an acyltransferase (ScAT10) sharing 80% identity with OsAT10 was identified and its expression in maize under the control of the maize ubiquitin promoter (*pZmUbi*) resulted in up to 160-fold increase in *p*CA-Ara in stems. Such increase is substantially higher than those observed in the stems of our *pSbUbi:AT10* sorghum lines (up to 42-fold increase) and previously published rice lines overexpressing *OsAT10* under the control of *pZmUbi* (4.6-fold increase) [19], which could be explained by differences in transgene expression levels and/or enzymatic specific activities. More generally, considering the potential for upgrading *p*CA to valuable chemicals, engineering approaches aiming at increasing *p*CA titers in bioenergy crops should be contemplated to increase biomass value [25].

Measurements of FA in sorghum lines expressing *OsAT10*, which was released from cell walls either as dimers and monomer upon mild alkaline hydrolysis, or as arabinose conjugate upon mild acid hydrolysis, did not reveal any major reductions compared to control plants. These results are in contrast with previous studies in which expression of AT10 transferases resulted in up to 60% and 90% reduction of FA-Ara in rice and maize, respectively [19, 22], while 21–27% reductions of FA released by dilute alkaline were observed in switchgrass [23]. Again, these observations could lie in variable *AT10* expression levels among engineered crops, but the impact of different native patterns of xylan acylation with *p*CA and FA between these plant species could be another explanation [7, 8]. From previous studies, it remains unclear how modification of *p*CA transfer activity onto xylan often results in modifications of xylan feruloylation levels, and vice-versa [17–23, 26]. In this work, higher amounts of certain diFAs were released from several sorghum *AT10* lines after mild alkaline hydrolysis (Fig. 3c), which could suggest an involvement of AT10 in feruloyl transferase activity, but an indirect positive effect of reduced lignin on the yield of diFA released cannot be excluded.

We observed reductions of lignin in stems from sorghum lines that express *OsAT10*, which is in contrast with previous measurements made in rice and switchgrass expressing the same transferase gene [19, 23]. It is unlikely that lignin reductions observed in the present study are the result of *p*-coumaroyl-CoA being partially rerouted towards xylan acylation at the expense of monolignol synthesis. Indeed, total cell wall-bound *p*CA remains unchanged in *AT10* trangenics compared to controls and its amount (ca. 8 mg/g CWR) is far below that of lignin (ca. 160 mg/g CWR). Moreover, it is unknown in sorghum whether feruloyl-CoA involved in xylan feruloylation and monolignol synthesis derives from *p*-coumaroyl-CoA, *p*CA, or both. For example, a metabolic route via direct hydroxylation of *p*CA catalyzed by *p*CA 3-hydroxylase (C3H) to produce caffeate (i.e., the precursor of FA) has been shown in *Brachypodium* and Arabidopsis, and *C3H* homologs have been identified in the sorghum genome [27]. Therefore, it would be interesting to examine in sorghum the possible contribution of C3H to feruloyl-CoA pools, xylan feruloylation, and lignification. Finally, an increase of cell wall-bound FA observed in sorghum lines overexpressing caffeoyl-CoA *O*-methyltransferase (CCoAOMT) suggests a participation of this enzyme in the synthesis of the feruloyl-CoA pool involved in xylan feruloylation [28]. Overall, more comprehensive data (e.g., from omics studies or radiolabeled tracing experiments) are needed to understand the mechanisms leading to lignin reductions and alteration of its monomeric composition in *AT10* engineered sorghum.

Finally, the improvements of saccharification efficiency observed in sorghum engineered with *OsAT10* are tentatively attributed to reduced lignin levels rather than drastic changes in the content of diFA forming bridges between xylan chains—a factor that also contributes to cell wall recalcitrance, but the level of FA esters specifically implicated in crosslinks with lignin needs to be further investigated [29]. Technoeconomic analysis and lifecycle assessment of biofuel production using sorghum as feedstocks and ionic liquid as solvent for biomass pretreatment have been recently conducted, especially regarding the benefits of using ensiled biomass [30]. The OsAT10 engineering approach could improve the economics of this process by reducing the concentration of ionic liquid and the amount of enzyme required to achieve optimal sugar yields from biomass.

## Conclusions

In this work, we validated in sorghum the effectiveness of expressing *OsAT10* to reduce biomass recalcitrance to enzymatic degradation. Unlike previous studies that correlated the improvements of biomass saccharification with changes in the amount of cell wall-bound FA and with increases in the ratio of *p*CA to FA, we show in the case of *OsAT10-*expressing sorghum that reduced recalcitrance is primarily the result of reduced lignin content. Nevertheless, other factors that contribute to cell wall digestibility such as the linkage types, degree of polymerization, and content of hydroxyl groups in lignin, as well as the degree of cellulose crystallinity or the interactions of xylan chains with lignin remain to be investigated and clarified. Our data also suggest a connection between xylan-bound *p*CA and lignification. In this regard, higher levels of xylan *p*-coumaroylation have been typically observed in organs containing less lignin such as leaves compared to stems in maize, *Brachypodium*, and miscanthus [7]. Finally, no obvious phenotypic alterations could be observed in engineered *OsAT10* sorghum under controlled growth conditions, but agronomic performances and validation of reduced recalcitrance will need to be assessed under field conditions.

## Methods

### Plant growth conditions and sampling

Plants were grown at the UC Berkeley South greenhouse Oxford facility between July and December 2019 with a minimum temperature set at 22 °C. T2 homozygous transgenic seeds and wild-type segregant seeds were germinated directly on soil (Sunshine mix #4, Sun Gro, Agawam, MA) in one-gallon pots and plants were grown until seeds reached the black layer stage (i.e., full physiological maturity). One tablespoon of Osmocote Plus 15-9-12 was added to the soil biweekly until the flowering stage. Watering was stopped at the end of the growing period and pots containing plants were allowed to dry for another 3 weeks. Growth parameters were measured at physiological maturity. For cell wall analyses, a 20-cm piece (starting 3 cm from the soil) from the main stem of each plant was harvested. Stem samples were further dried in an oven at 50 °C for 5 days and grinded into a fine powder using a Mixer Mill MM 400 (Retsch Inc., Newtown, PA) and stainless-steel balls.

### Design of the *pSbUbi:AT10* construct

The promoter sequence of the sorghum (*S. bicolor*) polyubiquitin gene was amplified by PCR using the primers listed in Additional file 1: Table S3 for level-0 cloning into the pBca9145 vector [31] using In-Fusion cloning (Takara Bio USA, Mountain View, CA). The DNA coding sequence of OsAT10 (UniProtKB/Swiss-Prot: Q69UE6.1) was codon-optimized for expression in sorghum and synthesized as a gene fragment by GenScript (Piscataway, NJ). The sequence contained flanking BsmBI restriction sites plus extra homologous sequences for cloning into pBca9145. The *pSbUbi:AT10* construct was obtained using the jStack cloning method [31] and the level-0 and level-1 intermediate plasmids are listed in Additional file 1: Table S4. Plasmid sequences are available at the Inventory of Composable Elements (ICE) source registry (http://public-registry.jbei.org).

### Sorghum transformation and genotyping

*Agrobacterium tumefaciens* was used to transform the grain sorghum variety Wheatland (*Sorghum bicolor* (L.) Moench). TaqMan Real-time PCR assays (Thermo Fisher Scientific, Waltham, MA) were performed on gDNA isolated from primary transformants using primers specific to the *nptII* and *OsAT10*^*opt*^ gene sequences to identify transformants with a single-copy event. Homozygous plants for the transgene and wild-type segregants were identified using the same approach in the T1 generation.

### RT-qPCR

Total RNAs were extracted from stems of 3-week-old plants in the T2 generation using the RNeasy Plant Mini Kit (Qiagen, Redwood City, CA) and cDNA synthesis was conducted using the SuperScript IV First-Strand Synthesis kit (Thermo Fisher Scientific, Waltham, MA) as previously described [32]. RT-qPCR was performed using 35 cycles consisting of 5 s at 95 °C for denaturation and 15 s at 60 °C for annealing and amplification. The relative quantification of *OsAT10* transcripts was calculated using the 2^−ΔCT^ method and normalized to the reference gene *PP2A* (NCBI Reference Sequence: XM_002453490.2) [33]. The results are the average from four biological replicates. RT-qPCR primers are listed in Additional file 1: Table S3.

### Biomass composition analysis

Ball-milled biomass was extracted as previously described [34]. Klason lignin and cell wall monosaccharides were determined on cell wall residues (CWR) using the standard NREL biomass procedure [35]. Glucose, xylose, and arabinose from biomass hydrolysates were measured by HPLC as previously described [36].

### Extraction of cell wall-bound aromatics

Total aromatics were released from 20 mg of CWR via mild alkaline hydrolysis as previously described [37]. FA and *p*CA were quantified using HPLC–ESI–TOF–MS analysis [38]. FA-Ara and *p*Ca-Ara were released from CWR as previously described using trifluoroacetic acid (TFA) hydrolysis [19]: 10 mg of CWR was incubated with 50 mM TFA at 100 °C for 2 h. Samples were centrifuged 5 min at 20,000×*g* and dried in vacuo to remove the TFA. Residues were resuspended in 50% (v/v) methanol:water and filtered using Amicon Ultra centrifugal filters (10,000 Da MW cutoff regenerated cellulose membrane; EMD Millipore, Billerica, MA, USA). FA-Ara and *p*Ca-Ara were analyzed using HPLC–ESI–TOF–MS [38]. For FA-Ara, *p*Ca-Ara and diFA isomers, the measured masses agreed with the expected theoretical masses within less than 5 ppm mass error. Fragmentation patterns were obtained by collision-induced dissociation (CID)–MS/MS using a quadrupole–TOF–MS with collision energies of 10 V.

### Lignin monomeric composition

Pyrolysis coupled with gas chromatography mass spectrometry was used to determine the S/G ratio, as previously described [39, 40]. Sub-samples of ca. 0.1 mg were pyrolyzed at 650 °C for 30 s using the pyroprobe 6200 (CDS Analytical, Oxford, PA) connected to a gas chromatography system (GC-2010 Plus, Shimadzu Scientific Instruments, Columbia, MD) using a Shimadzu SH-Rxi-5Sil MS column (30 m × 0.25 mm ID × 0.25 DF) attached to a mass spectrometer (GCMS-Q P2010, Shimadzu Scientific Instruments, Columbia, MD) system operated using He as carrier (1 mL min^−1^). The chromatograph was operated at a split ratio of 10 and the program was set at 50 °C for 1 min followed by ramping to 300 °C at 20 °C min^−1^ and finally maintained at 300 °C for 10 min. Released products of S and G origin were identified on the basis of their mass spectra using the NIST08 mass spectrum library and quantified from the chromatogram using the peak area.

### Saccharification assays

For pretreatment, a cholinium phosphate ionic liquid (IL) was prepared by mixing in an ice bath cholinium hydroxide and phosphoric acid in a 3:1 molar ratio, followed by lyophilization to semi solid consistency. Choline-based ILs have been recently proposed as solvents for biomass pretreatment [41]. Total biomass (200 mg) was mixed with 1.134 mL of an IL-deionized water solution (15:85, w/w) and autoclaved for 3 h at 121 °C. For saccharification, 620 µL of 1 M citrate buffer (pH 5.0) containing an enzyme mixture of Cellic® CTec3 and HTec3 (9:1 v/v) (Novozymes, Bagsværd, Denmark) was added to the pretreated biomass samples using a total enzyme loading of 10 mg g^−1^ biomass. Samples were rotated at 30 rpm at 50 °C for 72 h in a rotating incubator. Conditions used for saccharification assays (IL concentration, pretreatment temperature, and enzyme loadings) are considered to be suboptimal for total polysaccharide hydrolysis. For glucose and xylose measurements, hydrolysates were filtered using 0.2 µM PVDF filters (EMD Millipore, Billerica, MA, USA) and analyzed by HPLC as previously described [36].

## Supplementary Information

**Additional file 1: Figure S1.** Representative liquid chromatography–mass spectrometry (LC–MS) chromatograms obtained from analysis of TFA hydrolysates from CWR of wildtype (orange traces) and *pSbUbi:AT10* (blue traces) sorghum stems. Peaks corresponding to *p*CA-Ara (**a**) and FA-Ara (**b**) display major ions at m/z 295 and 325, respectively. Electrospray ionization collision-induced dissociation tandem mass (ESI–CID–MS/MS) spectra of *p*CA-Ara (**c**) and FA-Ara (**d**) are shown. Ions at *m*/*z* 265 and 235 correspond to the ^0,2^A_1_ and ^0,3^A_1_ arabinose cross-ring cleavage ions as previously reported (Quéméner and Ralet 2004). In addition, ions from deprotonated *p*-coumarate (*m*/*z* 163) and ferulate (*m*/*z* 193) are observed. **Figure S2.** Representative LC–MS chromatogram obtained from analysis of alkaline hydrolysates from CWR of wildtype sorghum stems. Seven peaks with ions at *m*/*z* 385 corresponding to diferulate isomers are observed. ESI–MS spectra from each peak are shown (bottom panels). Tentative diferulate chemical structures are based on the seven compounds previously identified in alkaline hydrolysates obtained from switchgrass and maize stalk cell walls (Ralph et al. 1994; Marita et al. 2003). **Table S1.** Growth parameters of wildtype and *pSbUbi:AT10* lines. Values in brackets are the SE from five biological replicates (*n* = 5). **Table S2.** Characteristics and relative molar abundances (%) of guaiacyl (G) and syringyl (S) lignin-specific pyrolysis products released from CWR of wildtype (WT) and pSbUbi:AT10 lines. Values in brackets are the SE from four biological replicates (*n* = 4). **Table S3.** List of primers used in this study. **Table S4.** List of plasmids used in this study.

## Data Availability

All the data supporting the conclusions of this article is included within the article and its additional files.

## Abbreviations

CaMV: Cauliflower mosaic virus
CoA: Coenzyme A
CWR: Cell wall residue
ESI–CID–MS/MS: Electrospray ionization collision-induced dissociation tandem mass spectrometry
FA-Ara: Feruloyl-arabinose
GC/MS: Gas chromatography/mass spectrometry
HPLC–ESI–TOF–MS: High performance liquid chromatography electrospray ionization and time-of-flight mass spectrometry
IL: Ionic liquid
*p*CA-Ara: *p*-Coumaroyl-arabinose
RT-qPCR: Real-time quantitative reverse transcription PCR
TFA: Trifluoroacetic acid
