# Supplementary material for: Overexpression of the rice BAHD acyltransferase AT10 increases xylan-bound p-coumarate and reduces lignin in Sorghum bicolor
Source: Biotechnol Biofuels. 2021 Nov 20;14:217. doi: 10.1186/s13068-021-02068-9 (PMC8606057; doi:10.1186/s13068-021-02068-9)
Supplement: Supplementary file 1 — Additional file 1: Figure S1. Representative liquid chromatography–mass spectrometry (LC–MS) chromatograms obtained from analysis of TFA hydrolysates from CWR of wildtype (orange traces) and pSbUbi:AT10 (blue traces) sorghum stems. Peaks corresponding to pCA-Ara (a) and FA-Ara (b) display major ions at m/z 295 and 325, respectively. Electrospray ionization collision-induced dissociation tandem mass (ESI–CID–MS/MS) spectra of pCA-Ara (c) and FA-Ara (d) are shown. Ions at m/z 265 and 235 correspond to the 0,2A1 and 0,3A1 arabinose cross-ring cleavage ions as previously reported (Quéméner and Ralet 2004). In addition, ions from deprotonated p-coumarate (m/z 163) and ferulate (m/z 193) are observed. Figure S2. Representative LC–MS chromatogram obtained from analysis of alkaline hydrolysates from CWR of wildtype sorghum stems. Seven peaks with ions at m/z 385 corresponding to diferulate isomers are observed. ESI–MS spectra from each peak are shown (bottom panels). Tentative diferulate chemical structures are based on the seven compounds previously identified in alkaline hydrolysates obtained from switchgrass and maize stalk cell walls (Ralph et al. 1994; Marita et al. 2003). Table S1. Growth parameters of wildtype and pSbUbi:AT10 lines. Values in brackets are the SE from five biological replicates (n = 5). Table S2. Characteristics and relative molar abundances (%) of guaiacyl (G) and syringyl (S) lignin-specific pyrolysis products released from CWR of wildtype (WT) and pSbUbi:AT10 lines. Values in brackets are the SE from four biological replicates (n = 4). Table S3. List of primers used in this study. Table S4. List of plasmids used in this study. [file 13068_2021_2068_MOESM1_ESM.pdf]

Overexpression of the rice BAHD acyltransferase AT10 increases xylan-bound *p*-coumarate and reduces lignin in *Sorghum bicolor*

Yang Tian, Chien-Yuan Lin, Joon-Hyun Park, Chuan-Yin Wu, Ramu Kakumanu, Venkataramana R. Pidatala, Khanh M. Vuu, Alberto Rodriguez, Patrick M. Shih, Edward E. K. Baidoo, Stephen Temple, Blake A. Simmons, John M. Gladden, Henrik V. Scheller, Aymerick Eudes

Additional file 1:

Figure S1. Representative liquid chromatography–mass spectrometry (LC–MS) chromatogram obtained from analysis of TFA hydrolysates from CWR of wild-type (orange traces) and *pSbUbi:AT10* (blue traces) sorghum stems. Peaks corresponding to *p*CA-Ara (**a**) and FA-Ara (**b**) display major ions at m/z 295 and 325, respectively. Electrospray ionization collision-induced dissociation tandem mass (ESI-CID-MS/MS) spectra of *p*CA-Ara (**c**) and FA-Ara (**d**) are shown. Ions at m/z 265 and 235 correspond to the ${}^{0,2}A_1$ and ${}^{0,3}A_1$ arabinose cross-ring cleavage ions as previously reported (Quéméner and Ralet, 2004). In addition, ions from deprotonated *p*-coumarate (m/z 163) and ferulate (m/z 193) are observed.

Figure S2. Representative LC–MS chromatogram obtained from analysis of alkaline hydrolysates from CWR of wildtype sorghum stems. Seven peaks with ions at m/z 385 corresponding to diferulate isomers are observed. ESI-MS spectra from each peak are shown (bottom panels). Tentative diferulate chemical structures are based on the seven compounds previously identified in alkaline hydrolysates obtained from switchgrass and maize stalk cell walls (Marita et al. 2003; Ralph et al. 1994).

Table S1. Growth parameters of wildtype (WT) and *pSbUbi:AT10* lines. Values in brackets are the SE from five biological replicates (*n* = 5).

Table S2. Characteristics and relative molar abundances (%) of guaiacyl (G) and syringyl (S) lignin-specific pyrolysis products released from CWR of wildtype and *pSbUbi:AT10* lines. Values in brackets are the SE from four biological replicates (*n* = 4).

Table S3. List of primers used in this study.

Table S4. List of plasmids used in this study.

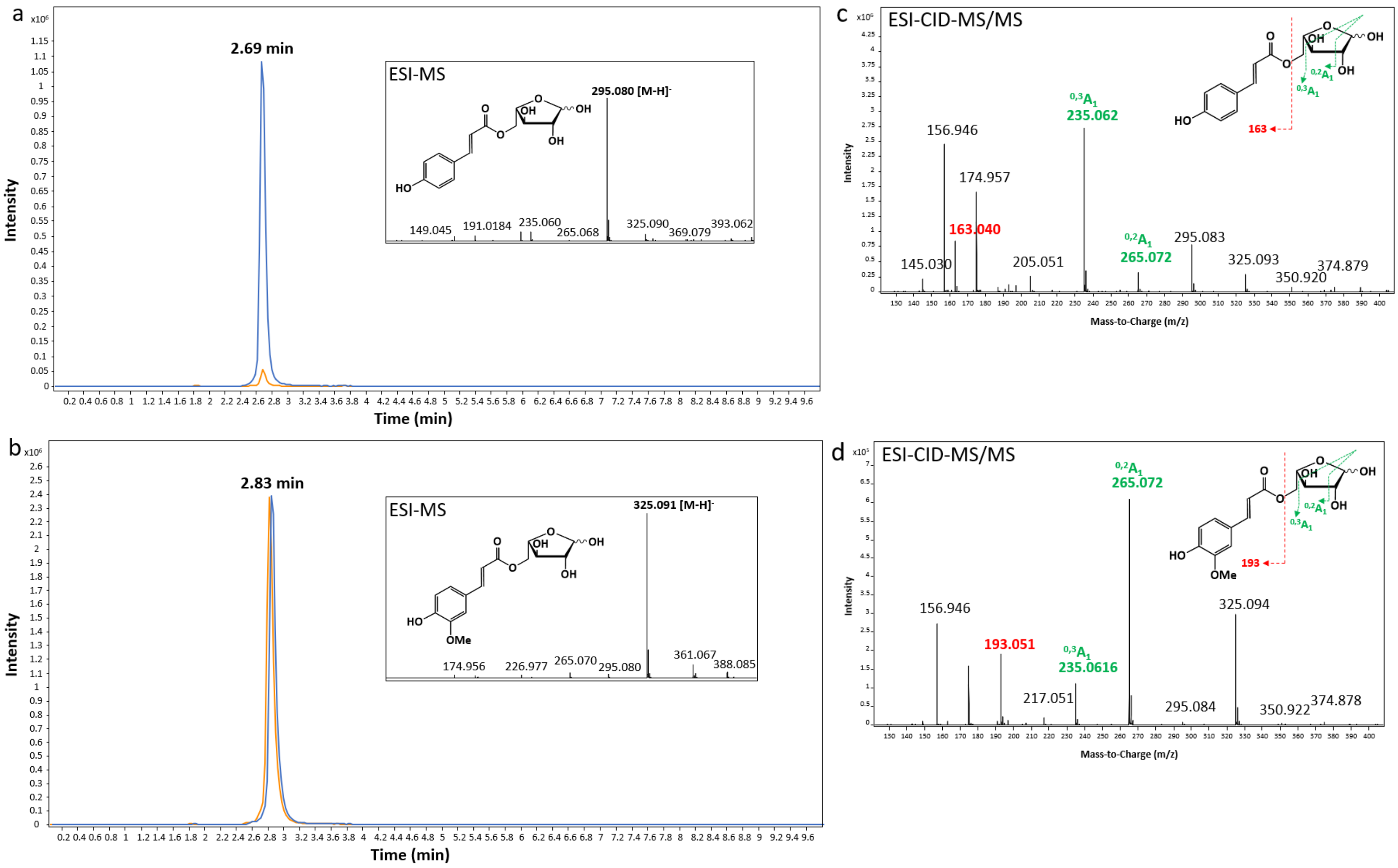

Figure S1. Representative liquid chromatography–mass spectrometry (LC–MS) chromatograms obtained from analysis of TFA hydrolysates from CWR of wildtype (orange traces) and *pSbUbi:AT10* (blue traces) sorghum stems. Peaks corresponding to *p*CA-Ara (**a**) and FA-Ara (**b**) display major ions at m/z 295 and 325, respectively. Electrospray ionization collision-induced dissociation tandem mass (ESI-CID-MS/MS) spectra of *p*CA-Ara (**c**) and FA-Ara (**d**) are shown. Ions at m/z 265 and 235 correspond to the $^{0,2}A_1$ and $^{0,3}A_1$ arabinose cross-ring cleavage ions as previously reported (Quéméner and Ralet, 2004). In addition, ions from deprotonated *p*-coumarate (m/z 163) and ferulate (m/z 193) are observed.

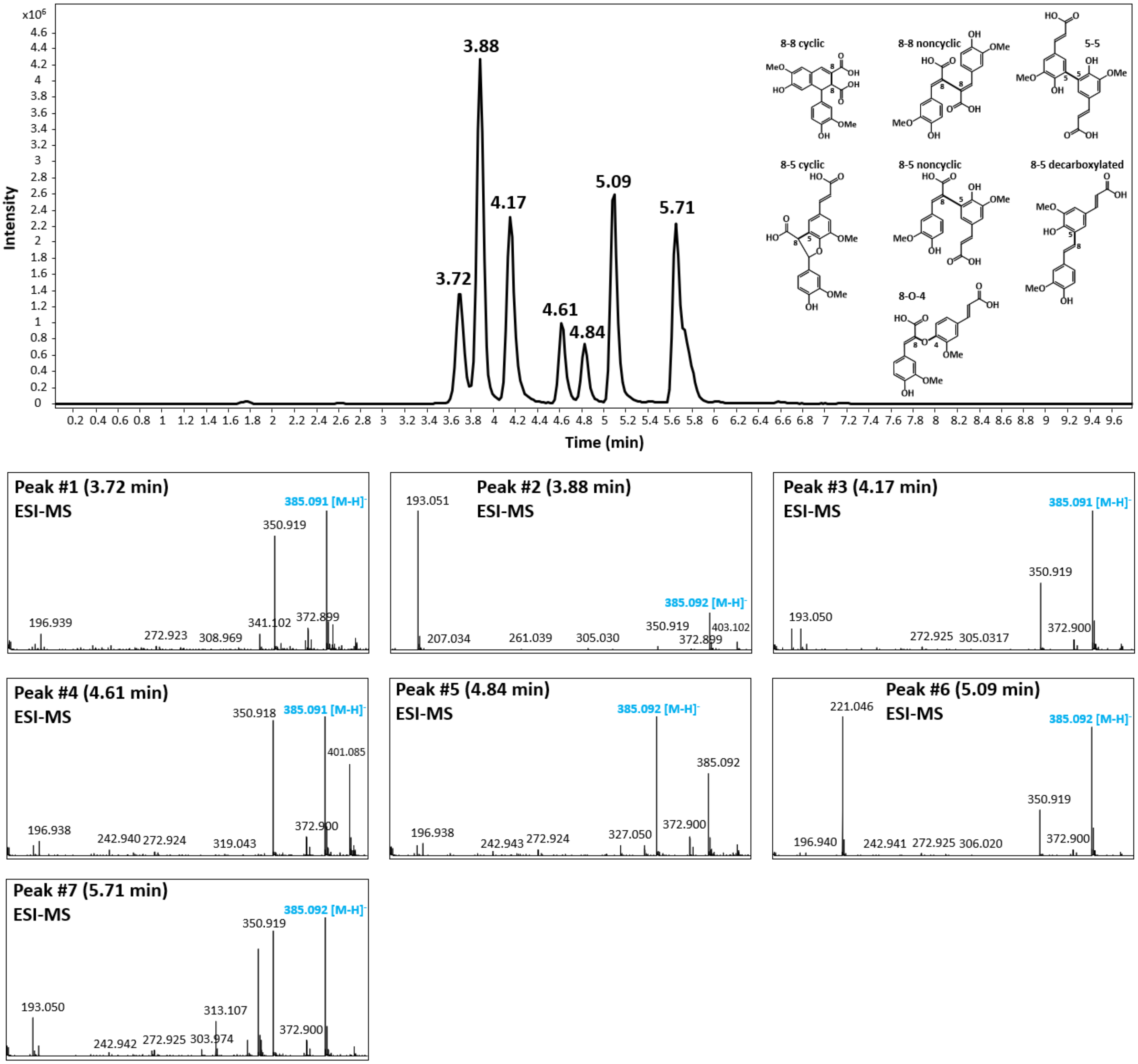

Figure S2. Representative LC–MS chromatogram obtained from analysis of alkaline hydrolysates from CWR of wildtype sorghum stems. Seven peaks with ions at m/z 385 corresponding to diferulate isomers are observed (elution times are indicated for each peak). ESI-MS spectra from each peak are shown (bottom panels). Tentative diferulate chemical structures are based on the seven compounds previously identified in alkaline hydrolysates obtained from switchgrass and maize stalk cell walls (Marita et al. 2003; Ralph et al. 1994).

Table S1. Growth parameters of wildtype and *pSbUbi:AT10* lines. Values in brackets are the SE from five biological replicates (n = 5).

	**Panicle emergence (days)**	**Number of flowering tillers (n)**	**Main tiller height (cm)**	**Stover dry weight (g)**	**1000-seed dry weight (g)**
Wildtype	55.2 (0.2)	5.0 (0.0)	88.1 (2.1)	102.7 (7.8)	38.8 (1.3)
pSbUbi:AT10 #15	55.2 (0.2)	5.0 (0.3)	**94.6 (2.3)***	113.7 (6.7)	40.4 (1.1)
pSbUbi:AT10 #23	55.6 (1.9)	5.2 (0.3)	91.9 (4.4)	122.4 (5.4)	39.6 (0.8)
pSbUbi:AT10 #34	55.6 (0.4)	4.8 (0.4)	93.4 (1.8)	**132.0 (11.4)***	41.0 (1.2)
pSbUbi:AT10 #47	55.0 (0.0)	4.0 (0.3)	**97.1 (2.2)****	89.5 (11.1)	37.0 (2.0)
pSbUbi:AT10 #50	56.2 (0.9)	4.2 (0.5)	94.1 (4.5)	124.2 (11.0)	39.6 (1.0)
pSbUbi:AT10 #54	55.2 (0.2)	4.2 (0.3)	87.7 (3.5)	104.5 (10.2)	37.8 (0.9)
pSbUbi:AT10 #57	53.8 (1.1)	4.0 (0.5)	85.7 (6.5)	104.0 (14.5)	42.0 (0.5)
pSbUbi:AT10 #73	55.4 (0.4)	5.8 (0.8)	**77.5 (2.1)****	97.5 (11.0)	36.8 (1.3)

Asterisks indicate a significant difference from the wildtype using the unpaired Student's t-test (P < 0.05; *P < 0.1).**

Table S2. Characteristics and relative molar abundances (%) of guaiacyl (G) and syringyl (S) lignin-specific pyrolysis products released from CWR of wildtype (WT) and *pSbUbi:AT10* lines. Values in brackets are the SE from four biological replicates (n = 4).

Compound name	Origin	Formula	MW	Main mass fragments	Elution time (min)	*pSbUbi:AT10* lines								WT
						#15	#23	#34	#47	#50	#54	#57	#73	
2-methoxyphenol	G	$C_7H_8O_2$	124	81, 109, 124	8.4	18.1 (2.8)	23.1 (1.8)	21.6 (1.0)	19.9 (1.7)	16.5 (2.1)	14.6 (1.1)	16.0 (2.8)	18.7 (0.6)	14.5 (0.5)
2-methoxy-4-methylphenol	G	$C_8H_{10}O_2$	138	95, 123, 138	9.3	11.9 (1.5)	8.9 (1.7)	10.8 (1.9)	10.1 (2.3	15.4 (1.7)	11.0 (1.1)	7.5 (2.8)	7.0 (1.3)	8.8 (1.1)
4-ethyl-2-methoxyphenol	G	$C_9H_{12}O_2$	152	125, 137, 152	9.9	5.3 (0.2)	18.4 (1.8)	25.0 (1.8)	17.4 (1.5)	13.5 (0.7)	19.7 (1.0)	16.8 (2.8)	18.9 (0.9)	15.1 (0.6)
2,6-dimethoxyphenol	S	$C_8H_{10}O_3$	154	65, 93, 139, 154	10.5	20.0 (1.3)	17.1 (1.3)	17.0 (0.3)	20.3 (0.5)	20.2 (0.6)	19.2 (1.5)	20.7 (2.8)	21.2 (0.6)	19.7 (1.5)
3,5-dimethoxy-4-hydroxytoluene	S	$C_9H_{12}O_3$	168	125, 153, 168	11.1	7.3 (0.4)	4.0 (0.3)	5.1 (0.7)	5.4 (0.7)	5.9 (0.1)	5.1 (1.0)	5.9 (2.8)	6.4 (0.7)	8.1 (0.6)
2-methoxy-4-(1-propenyl)phenol	G	$C_{10}H_{12}O_2$	164	55, 77, 91, 103, 164	11.3	20.1 (0.9)	14.4 (0.5)	11.5 (0.6)	13.6 (0.5)	16.4 (1.7)	16.5 (1.6)	16.1 (2.8)	12.0 (1.2)	18.9 (0.8)
4-allyl-2,6-dimethoxyphenol	S	$C_{11}H_{14}O_3$	194	167, 179, 194	12.2	4.7 (0.1)	5.3 (1.1)	4.2 (0.2)	5.5 (1.0)	5.2 (0.4)	6.0 (0.4)	6.6 (2.8)	5.8 (0.1)	5.8 (0.5)
2,6-dimethoxy-4-(2-propenyl)phenol	S	$C_{11}H_{14}O_3$	194	77, 91, 194	12.9	7.8 (0.5)	8.9 (1.3)	4.7 (0.4)	7.9 (0.8)	6.9 (0.6)	7.9 (0.6)	10.4 (2.8)	10.0 (0.5)	9.0 (0.5)
%G						60.3 (1.3)	64.8 (1.5)	69.0 (0.5)	61.0 (1.0)	61.8 (0.2)	61.8 (1.5)	56.5 (0.6)	56.6 (1.1)	57.4 (0.8)
%S						39.7 (1.3)	35.2 (1.5)	31.0 (0.5)	39.0 (1.0)	38.2 (0.2)	38.2 (1.5)	43.5 (0.6)	43.4 (1.1)	42.6 (0.8)

Table S3: Primers used in this study

Primer name	Purpose / Target	Sequence (5'-3')
BsaI-pSbUbi-Fw	Part isolation / *pSbUbi* gDNA	cgctaaggatgatttctggaattcggtctcTggagCACTAATCCACCAATACATAA
BsaI-pSbUbi-Rv		cagctcgagttaggatccggtctcAcattCTGCAAACGTCAACAAGCAAAAGC
OsAT10-qPCR-Fw	RT-qPCR / *OsAT10* cDNA	ACCCCTTGTGAATTGCGACC
OsAT10-qPCR-Rv		CTTGGTCACACAGGAGGCGA
PP2A-qPCR-Fw	RT-qPCR / *PP2A* cDNA	AACCCGCAAAACCCCAGACTA
PP2A-qPCR-Rv		TACAGGTCGGGCTCATGGAAC

Table S4. List of plasmids used in this study

Construct name	Level	Backbone	Description	JBEI ICE ID
pSbUbi:AT10	2	pPMS074 (Lin et al., 2021)	*tOCS-Kan^R-pSbUbi:OsAT10^{opt}-tNOS*	JBx_090067
pSbUbi:AT10-Lv1	1	pPMS028 (Shih et al., 2016)	Level-1 construct obtained with level-0 parts: {L_tOCS-Kan^R}, {P_SbUbi}, {OsAT10^{opt}}, and {T_tNOS}	JBx_150615
{L_tOCS-Kan^R}	0	pBca9145 (Shih et al., 2016)	*Agrobacterium* octopine synthase terminator and plant hygromycin selectable marker, primary linker	JBx_065723
{P_SbUbi}	0		Promoter of the polyubiquitin gene from sorghum (NCBI Reference Sequence: NC_012879.2)	JBx_092922
{OsAT10^{opt}}	0		AT10 BAHD acyltransferase gene from rice (GenBank: BAD33123.1), codon-optimized	JBx_090024
{T_tNOS}	0		*Agrobacterium* nopaline synthase terminator	JBx_042266

References

Marita JM, Vermerris W, Ralph J, Hatfield RD. Variations in the cell wall composition of maize brown midrib mutants. J Agric Food Chem. 2003;51:1313–21.

Quéméner B, Ralet MC. Evidence for linkage position determination in known feruloylated mono- and disaccharides using electrospray ion trap mass spectrometry. J Mass Spectrom. 2004;39:1153–60.

Ralph J, Quideau S, Grabber JH, Hatfield RD. Identification and synthesis of new ferulic dehydrodimers present in grass cell walls. J Chem Soc Perkin Trans. 1994;1:3485–98.
